# A Dynamical Systems-Based Hierarchy for Shannon, Metric and Topological Entropy

**DOI:** 10.3390/e21100938

**Published:** 2019-09-25

**Authors:** Raymond Addabbo, Denis Blackmore

**Affiliations:** 1 Department of Arts and Sciences, Vaughn College of Aeronautics and Technology, Flushing, NY 11369, USA; 2 Department of Mathematical Sciences and Center for Applied and Computational Mathematics, New Jersey Institute of Technology, Newark, NJ 07102-1982, USA; denis.l.blackmore@njit.edu

**Keywords:** topological entropy, Shannon entropy: metric entropy, Bernoulli scheme

## Abstract

A rigorous dynamical systems-based hierarchy is established for the definitions of entropy of Shannon (information), Kolmogorov–Sinai (metric) and Adler, Konheim & McAndrew (topological). In particular, metric entropy, with the imposition of some additional properties, is proven to be a special case of topological entropy and Shannon entropy is shown to be a particular form of metric entropy. This is the first of two papers aimed at establishing a dynamically grounded hierarchy comprising Clausius, Boltzmann, Gibbs, Shannon, metric and topological entropy in which each element is ideally a special case of its successor or some kind of limit thereof.

## 1. Introduction

Entropy, which can among a variety of other things, be roughly viewed as a measure of uncertainty (cf. [1]), has been and remains a fascinating and still not completely understood concept with an amazing range of applications, including quantum and ecological systems [2]. We have discovered that many mathematical, scientific and engineering colleagues share our abiding curiosity about rigorous connections among the myriad definitions of entropy. For example, what if any is the relationship between classical Clausius entropy and topological entropy or even Shannon entropy? There have been several extensive investigations of various types of entropy including historical accounts of the relevant developments and informative investigations of the linkages among the various forms of entropy such as in [3,4,5,6,7,8,9,10,11,12,13], but they all appear to be somewhat lacking in terms of identification of truly compelling unifying themes for the multifarious definitions.

Our intention in this and a subsequent paper is to provide a rigorous partial answer to this question by proving that there is a dynamical systems thread connecting all of the following definitions of entropy leading, with the possible addition of some mild assumptions, to the hierarchy

Clausius⟵Gibbs⟵Boltzmann⟵Shannon⟵metric⟵topological

By rigor as used here, we mean that any connections and special case identifications shall be proved. In this paper, we shall establish a dynamical systems thread connecting Shannon, metric and topological entropy; in a forthcoming paper we hope to complete the hierarchy by elucidating the connections among the first three of the above and their link to the last three. In a related note, we recommend a recent interesting functorial connection among these three entropies introduced by Delvenne [14].

The material to be presented is organized as follows. In the interest of completeness, we present the definitions and some basic properties of topological, metric and Shannon entropy. Then, in Section 3, we prove that metric entropy is a special case of topological entropy if one adds just a few assumptions. Equality of metric and topological entropy can and shall be established in the following two forms: Constructively, namely by showing that certain measurable dynamical systems can be given a topology, which might be quite far removed from some of the more usual types, for which the two entropies in question are equal and by comparison, where one can sometimes determine when a given topology on a measurable dynamical system yields equality of the entropies. Next, in Section 4, we show that Shannon entropy is special case of metric entropy when formulated in a certain dynamical context. In particular, one can frame the underlying information theory foundation as a measurable dynamical system in which the metric and Shannon entropies are equal. As an example, we show how the result for a binary alphabet can be obtained essentially from scratch, in stark contrast to the general result due mainly to Kolmogorov and Sinai that involves very deep and extensive analysis. Finally, in Section 5 we summarize some of the conclusions reached and outline our plans for related future work on extending the hierarchy for entropies.

## 2. Topological, Metric and Shannon Entropy

In this section we define topological, metric and Shannon entropy, list some of their properties, and describe several basic relationships among them. We begin with the topological entropy of a discrete dynamical system on a topological space.

### 2.1. Topological Entropy

Let *X* be a nonempty Hausdorff topological space with topology T (comprising the open subsets of the space). A *topological discrete (semi-) dynamical system* (or just *discrete dynamical system* for short) is a continuous action of the form

$$F:N^{*}\times X\to X,$$

where $F\left(n,x\right):=f^{n}\left(x\right)$, f:X→X is a continuous map, $f^{n}$ is the *n*-th iterate of the map under composition ∘, with the convention that $f^{0}$ is the identity map $id=id_{X}$ and $N^{*}$ is the abelian semigroup {0}∪N under addition. There is a standard nomenclature (see, for example [15]) that includes such definitions as (positive semi-) *orbits* $O\left(x\right):=\{f^{n}\left(x\right):n\in$$N^{*}\}$ and *fixed points* where f(x)=x, so that O(x)={x}. For convenience, we shall denote the dynamical system by $D:=\left(f,X\right)$.

Assuming *X* to be compact, Adler et al. [16] defined the topological entropy of the dynamical system as follows: If U={U} is any open covering of X, the minimum cardinality N(U) over all subcoverings must be finite owing to the compactness of *X*. Now, if U and V={V} are two open coverings of *X*, so is their *common refinement*

$$U\vee V:=\left\{U\cap V:(U,V)\in U\times V\right\},$$

every set of which is contained in a set of both U and V, with the refinement relation being typically denoted as U,V≺U∨V. By iterating refinement, for each n∈N we obtain the open covering $U^{n}:=U\vee f^{-1}\left(U\right)\vee \cdots \vee f^{-n+1}\left(U\right)$ for which it can readily be shown that

(1) $$h_{T}\left(U,f\right)=\lim_{n\to \infty }\frac{\log N\left(U^{n}\right)}{n}=\inf\left\{\frac{\log N\left(U^{n}\right)}{n}:n\in N\right\}$$

exists. Then, the *topological entropy* is defined as

(2) $$h_{T}\left(f\right):=\sup\left\{h_{T}\left(U,f\right):U\in OC\left(X\right)\right\},$$

where OC(X) is the set of all open coverings of *X*.

There is also another (metric-based) definition of topological entropy (see Bowen [17], Dinaburg [4,5] and [18]), which for compact metric spaces is equivalent to the Adler–Konheim–McAndrew definition above.

It should be remarked that although we have confined ourselves to compact spaces, it is possible to extend the definition of topological entropy to noncompact spaces (cf. [19,20,21]). There is an analogous definition of topological entropy for *continuous dynamical systems* that applies to continuous (usually continuously differentiable), which we include for possible future reference. It applies to continuous actions of the type

Φ:R×X→X,

where R is the topological abelian group of real numbers with respect to addition. These actions arise naturally from solutions of autonomous systems of ordinary differential equations satisfying conditions guaranteeing global existence and uniqueness. There is the usual notation for these systems, which includes for example, the positive semiorbit through *x* defined as $O_{+}\left(x\right):=\left\{\varphi_{t}\left(x\right):=\Phi \left(t,x\right):t\geq 0\right\}$ The associated time-1 map $\varphi_{1}$, which generates a discrete dynamical system, is defined as $\varphi_{1}\left(x\right):=\Phi \left(1,x\right)$, and the topological entropy of the continuous dynamical system is

(3) $$h_{T}\left(\Phi \right):=h_{T}\left(\varphi_{1}\right)=\sup\left\{h_{T}\left(U,\varphi_{1}\right):U\in OC\left(X\right)\right\}=\sup\left\{h_{T}\left(U,f\right):U\in OC\left(X\right)\right\}.$$

Finally, we note in passing that the topological entropy in both the discrete and continuous cases can be intuitively viewed as the exponential growth in distinct orbits with discrete or continuous time, respectively.

#### Some Properties of Topological Entropy

Obviously, the topological entropy is nonnegative, and one can use the basic definitions and a bit of straightforward analysis (as in [15,16]) to prove the following properties:

(TE1) $h_{T_{X}}\left(f\right)=h_{T_{Y}}\left(g\right)$ if f:X→X and g:Y→Y are *topologically conjugate*(there is a homeomorphism φ:X→Y such that g∘φ=φ∘f)
(TE2) $h_{T}\left(f\right)\geq h_{T_{Y}}\left(f_{|Y}\right)$ if Y⊂X is closed and *f*-invariant and $T_{Y}$ is the subspace topology of T on *Y*, where $f_{|Y}$ denotes the restriction of *f* to *Y*
(TE3) $h_{T}\left(f\right)=h_{T}\left(f^{-1}\right)$ if *f* is a homeomorphism
(TE4) $h_{T}\left(f^{n}\right)=nh_{T}\left(f\right)$ if n∈N
(TE5) $h_{T_{X}\times T_{Y}}\left(f\times g\right)=h_{T_{X}}\left(f\right)+h_{T_{Y}}\left(g\right)$
(TE6) If a sequence $\left\{U_{n}\right\}$ of open covers is *refining* - meaning that $U_{n}≺U_{n+1}$ for every n∈N and for every open cover V of *X* there exists an *n* such that V≺$U_{n}$ - then $\left\{h_{T}\left(U_{n},f\right)\right\}$ is a nondecreasing sequence, with $h_{T}\left(f\right)=\lim_{n\to \infty }h_{T}\left(U_{n},f\right)=\sup\left\{h_{T}\left(U_{n},f\right):n\in N\right\}$.

### 2.2. Kolmogorov–Sinai Metric Entropy

A discrete *measurable dynamical system* consist of a nonempty set *X*, a σ-algebra of μ-measurable subsets M of *X*, where μ is a probability measure (with μ(X)=1) and a measurable function f:X→X (satisfying $f^{-1}\left(A\right)\in M$ whenever A∈M) such that μ is *f*-invariant, which means that $\mu \circ f^{-1}=\mu$ on M. The iterates of *f* obviously also define a discrete dynamical system, with the usual notions of orbits, fixed points and the like.

The *metric*(Kolmogorov–Sinai or just *K–S*) *entropy* requires a number of introductory elements for its definition. A *measurable partition* of *X* is a finite, pairwise-disjoint sequence $P=\{Q_{1},\ldots ,Q_{m}\}$ of μ-measurable sets of positive measure such that $X={⋃}_{k=1}^{m}Q_{k}$, and it is convenient to denote the set of all such measurable partitions of *X* as P(X). The *entropy of a measurable partition* is defined as

(4) $$H\left(P\right):=-\sum_{Q\in P}\mu \left(Q\right)\log\mu \left(Q\right).$$

As with open coverings in the definition, we can define a common refinement of measurable partitions P and $\tilde{P}$ as

$$P\vee \tilde{P}:=\left\{Q\cap \tilde{Q}:\left(Q,\tilde{Q}\right)\in P\times \tilde{P}\right\},$$

for which the refinement relation is analogously denoted by $P,\tilde{P}≺P\vee \tilde{P}$.

Iterating refinement in a manner analogous to that used for topological entropy, we define measurable partitions associated to *f* of the form

$$P^{n}:=P\vee f^{-1}\left(P\right)\vee \cdots \vee f^{-n+1}\left(P\right),$$

and define

(5) $$H\left(P,f\right):=\lim_{n\to \infty }\frac{H\left(P^{n}\right)}{n},$$

where the limit can be readily shown to exist. Then, we define the metric entropy as

(6) $$h_{\mu }\left(f\right):=\sup\left\{H\left(P,f\right)\right\}:P\in P\left(X\right)\},$$

and take note of the strong similarity with the definition of topological entropy.

It should also be mentioned that just as for the case of topological entropy, we can define metric entropy for continuous measurable dynamical systems using the time-1 map of the flow as in (3). As with the topological entropy, there are intuitive characterizations of metric entropy, two of which are the following: The exponential growth rate of typical orbits; and the maximum of the rate of extractable information.

#### Basic Properties of K–S Entropy

The nonnegativity of K–S entropy is clear from its definition, and we have the following basic properties that are clearly analogs of those for topological entropy, with the last of which following from the concavity of −xlogx (see [10,15,22,23,24,25]).

(ME1) $h_{\mu }\left(f\right)=h_{\nu }\left(g\right)$ if f:X→X and g:Y→Y are *metrically conjugate*(there is a measurable bijection φ:X→Y such that g∘φ=φ∘f and $\nu =\mu \circ \varphi^{-1}$)
(ME2) $h_{\mu }\left(f\right)\geq h_{\mu_{|Y}}\left(f_{|Y}\right)$ if Y⊂X is measurable and *f*-invariant and $\mu_{|Y}$ is the restriction of μ to *Y*, where $f_{|Y}$ denotes the restriction of *f* to *Y*
(ME3) $h_{\mu }\left(f\right)=h_{\mu }\left(f^{-1}\right)$ if *f* is a measurable bijection
(ME4) $h_{\mu }\left(f^{n}\right)=nh_{\mu }\left(f\right)$ if n∈N
(ME5) $h_{\mu \times \nu }\left(f\times g\right)=h_{\mu }\left(f\right)+h_{\nu }\left(g\right)$
(ME6) If a sequence $\left\{P_{n}=\left\{Q_{1},\ldots ,Q_{m_{n}}\right\}\right\}$ in P(X) is *refining* - meaning that $P_{n}≺P_{n+1}$ for every n∈N and $∥P_{n}∥:=\max\left\{\mu \left(Q_{k}\right):1\leq k\leq m_{n}\right\}\to 0$ as n→∞ - then $\left\{h_{\mu }\left(U_{n},f\right)\right\}$ is a nondecreasing sequence with $h_{\mu }\left(f\right)=\lim_{n\to \infty }h_{\mu }\left(U_{n},f\right)=\sup\left\{h_{\mu }\left(U_{n},f\right):n\in N\right\}$.

### 2.3. Shannon Entropy

In contrast with topological and metric entropy, Shannon entropy (see [26,27]) has an information theory rather than a dynamical system foundation. The basic elements can be distilled as follows: Let $S:=\{s_{1},\ldots ,s_{m}\}$ be a nonempty finite set of symbols or messages (sometimes referred to as the alphabet) with a discrete probability *p* assigned to each, such that $p(s_{i})\geq 0$ for all 1≤i≤m and $p\left(s_{1}\right)+\cdots +p\left(s_{m}\right)=1.$ Then, the *Shannon entropy* of the message ensemble

(7) $$H\left(S\right):=-\sum_{i=1}^{m}p\left(s_{i}\right)\log p\left(s_{i}\right)$$

is just the average or expected value of the information content of the message ensemble *X*, which is very much like the entropy of a measurable partition used in the definition of metric entropy.

#### Several Properties of Shannon Entropy

It follows from (7) that the Shannon entropy is nonnegative, and there are several other readily verified properties such as:

(SE1) $H\left(S\right)=H\left(\tilde{S}\right)$ if there is a bijection $\varphi :S\to \tilde{S}$ such that g∘φ=φ∘f and $\tilde{p}=p\circ \varphi^{-1}$
(SE2) $H\left(S\right)\geq H\left(\tilde{S}\right)$ if $\tilde{S}\subset S$
(SE3) $H\left(S\times \tilde{S}\right)=H\left(S\right)+H\left(\tilde{S}\right)$ if *S* and $\tilde{S}$ are independent (prob$\left(s_{i},{\tilde{s}}_{j}\right)=p\left(s_{i}\right)\tilde{p}\left({\tilde{s}}_{j}\right)$ for all i,j)
(SE4) H(S)=logn is maximized when $p\left(s_{1}\right)=\cdots =p\left(s_{n}\right)=1/n.$

### 2.4. A Relationship among the Entropies

Here we shall describe a well known relationship between topological and metric entropy obtained by Dinaburg [4,5], Goodman [8], Goodwyn [9] and Misiurewicz [28] using variational techniques (see also [12,13,15]), and often referred to as the *variational principle*. Let f:X→X be a continuous map on a compact metric space *X* with topology T, which defines a discrete dynamical system that we represent by the triple $\left(f,X,T\right)$. To establish the topological-metric entropy relation, it is convenient to define

(8) $$M\left(X\right):=\left\{\mu :\mu \;\mathrm{an}\;\mathrm{invariant}\;\mathrm{Borel}\;\mathrm{probability}\;\mathrm{measure}\;\mathrm{on}\;\mathrm{the}\;\sigma -\mathrm{algebra}\;M_{\mu }\;\mathrm{of}\;\mathrm{subsets}\;\mathrm{of}\;\;X\right\},$$

where Borel means that $T\subset M_{\mu }$ for all μ∈M(X). Then we have the following results, which is proved very efficiently in [28]:

**Theorem** **1.** *If X is a compact metric space with topology T and f:X→X is a continuous map, then*

(9) $$h_{T}\left(f\right):=\sup\left\{h_{\mu }\left(f\right):\mu \in M\left(X\right)\right\}.$$

## 3. When Topological Entropy Equals Metric Entropy

In this section we shall prove there is a topology on numerous discrete measurable dynamical systems (DMDS) of interest such that the corresponding topological entropy is equal to the K–S entropy. Let $\left(f,X,M,\mu \right)$ be a measurable dynamical system having metric entropy $h_{\mu }\left(f\right)$. We shall show that in certain cases there is a topology T on *X* for which f:X→X is continuous and $h_{T}\left(f\right)=h_{\mu }\left(f\right)$. One interesting example, which is an example of type (F2) that concerns Conjecture 3 of [16], which has since been proven (see [17,18,21,29]); namely, if *X* is a compact separable topological group, with topology T, and f:X→X is a continuous automorphism, then the Haar measure μ is *f*-invariant, and $h_{T}\left(f\right)=h_{\mu }\left(f\right)$. What we also have in mind is a Constructive-type of problem that involves defining a topology on a given DMDS, with no pre-specified topology, such that the topological and K-S entropies coincide and this is where we shall begin.

### 3.1. Constructive-Type Equality

The question is when can the phase space *X* of a given DMDS can be endowed with a compact metric topology T such that $h_{T}\left(f\right)=h_{\mu }\left(f\right)$. Our main result, which follows directly from the Jewett–Krieger theorem (cf. [3,30,31]), seems not to have appeared in the literature.

**Theorem** **2.** *Let $D=\left(f,X,M,\mu \right)$ be a measurable dynamical system, with f:X→X bijective and ergodic. Then, there exists a compact metric topology T on X such that $h_{T}\left(f\right)=h_{\mu }\left(f\right)$.*

**Proof.**  The Jewett–Krieger theorem implies that there is a compact metric space $\hat{X}$ with topology $\hat{T}$ with a continuous map $\hat{f}:\hat{X}\to \hat{X}$, $\hat{f}$-invariant Borel measure $\hat{\mu }$, and a sigma-algebra $\hat{M}$ of $\hat{\mu }$-measurable subsets of $\hat{X}$ defining a measure-theoretic dynamical system $\hat{D}=\left(\hat{f},\hat{X},\hat{M},\hat{\mu }\right)$ with the following properties:

(i) There is a *measure-theoretic dynamical system embedding* $\Phi :D\to \hat{D}$, which means there is an injective map $\phi :X\to \hat{X}$ such that $\hat{f}\circ \phi =\phi \circ f$ and A∈M implies that $\phi \left(A\right)\in \hat{M}$, with $\hat{\mu }\left(\phi (A)\right)=\mu \left(A\right).$
(ii) If ${\hat{\mu }}_{*}$ is the restriction of $\hat{\mu }$ to ϕ(X) and ${\hat{T}}_{*}$ is the subspace topology induced by $\hat{T}$ on ϕ(X), then $h_{\hat{T}}\left(\hat{f}\right)=h_{{\hat{\mu }}_{*}}\left(\hat{f}\right)$.

The choice of the desired topology can be inferred readily from the above; namely, we define T to be the topology induced by the injection $\phi :X\to \hat{X}$. As is well known, this topology T is simply that which is generated by the set $\left\{\phi^{-1}\left(V\right):V\in \hat{T}\right\}$. Hence, owing to the properties described above, the systems are rendered both topologically and metrically conjugate on ϕ(X). This completes the proof since it follows that (ii) implies $h_{T}\left(f\right)=h_{\mu }\left(f\right)$. □

Although the above is a simple corollary of the Jewett–Krieger result, the proof of the theorem itself is quite long and deep. This suggests that there may be a more direct proof of Theorem 1, which is something worth investigating.

### 3.2. Comparison-Type Equality

Since we start with a choice of the topology, the construction involves the possibility of noncompactness for which there are approaches in the literature such as [18,19,20,21,29,32,33]. However, we avoid this issue by selecting a compact topology for *X*.

We begin with the construction of the topology T for the phase space *X* of the DMDS $D:=\left(f,X,M,\mu \right)$ that we hope yields the equality of the two entropies. A rather natural choice for T is the topology $T_{0}\left(\mu \right)$ generated by the sets in all possible measurable partitions of *X* comprising subsets of positive measure, but this has inherent problems, not the least of which concerns compactness. Certainly, $\left(X,T_{0}\left(\mu \right)\right)$ is not *a priori* compact, nor as it turns out is it suitable for proving that the corresponding topological and given metric entropies are equal. Consequently, we must be more restrictive in the definition of the chosen topology as well as the DMDS.

First, we deal with the choice of topology and then describe some of the fundamental properties of the DMDS vis a vis the topology that we will use to guarantee the equality of the metric and topological entropies. Our first assumption is that *X* can be compactly embedded in a metric space *Y* with metric *d*, so it may be considered as a compact metric space with metric *d* restricted to *X*, with corresponding metric topology $T_{d}$. This immediately takes care of the question of compactness, for example, and provides other desirable properties associated with metric topologies. Moreover, it applies to any finite-dimensional, smooth compact manifold. Another convenient assumption connecting the topology $T_{d}$ and D is the following: The measure is Borel with respect to the topology; i.e., $T_{d}$ ⊂M and so all open sets are μ-measurable. It is worth noting that compact smooth manifolds with the usual types of measures typically satisfy these two assumptions. We shall also find it convenient to assume that the map *f* is continuous with respect to $T_{d}$, which implies that the map is also proper; i.e., the preimage of compact sets are compact.

#### 3.2.1. Definitions and Additional Notation

The above assumptions are basic and conveniently serve our needs, but several more subtle properties are required to obtain equality of entropies, which are best delineated by introducing some additional simplifying notation in the context of the assumptions that have so far been made. For example, the following notion shall prove useful.

**Definition** **1.** *A (finite) open cover $U=\{U_{1},\ldots ,U_{m}\}$ of X is minimal with respect to a measurable partition $P=\{Q_{1},\ldots ,Q_{m}\}$ of X if $Q_{k}\subset U_{k}$ for every 1≤k≤m and no proper subset of U is a covering of X. For convenience, we denote this as U-min-P.*

**Definition** **2.** *A measurable partition $P=\{Q_{1},\ldots ,Q_{m}\}$ is an α-β (with 0<α<β) partition of X with respect to $T_{d}$ and denoted as $P\left(\alpha ,\beta \right)$ if the following properties obtain for all the elements: (i) $\mu (Q_{k})>0$; (ii) $Q_{k}$ is connected in the topology $T_{d}$; (iii) the diameter $d(Q_{k})<\beta$; and (iv) there is at least one point $x\in Q_{k}$ such that the (closed ball) ${\bar{B}}_{\alpha }\left(x\right):=\left\{y\in X:d\left(x,y\right)\leq \alpha \right\}$, corresponding to the open ball $B_{\alpha }\left(x\right):=\left\{y\in X:d\left(x,y\right)<\alpha \right\}$, is contained in $Q_{k}$. We denote the set of all α-β partitions as $P_{\alpha \beta }\left(X\right)$.*

**Definition** **3.** *The DMDS D is of L-type for $T_{d}$ if it satisfies the following properties: (i) $\mu (B_{\alpha }\left(x\right)>0$ for every α>0; (ii) $\mu (B_{\alpha }\left(x\right)\to 0$ as α→0; (iii) for every element $Q_{k}$ of an α-β partition of X and ϵ>0 there is a connected open set $U_{k}\left(\epsilon \right)$ such that $Q_{k}\subset U_{k}\left(\epsilon \right),$$\mu \left(U_{k}\left(\epsilon \right)\setminus Q_{k}\right)<\epsilon$ and $d\left(U_{k}\left(\epsilon \right)\right)<\beta +\epsilon$; (iv) $d\left(x,\partial U_{k}\left(\epsilon \right)\right)<\epsilon /2$ for every $x\in \partial Q_{k}$ and (v) $U_{k}\left(\epsilon_{1}\right)\subset$$U_{k}\left(\epsilon_{2}\right)$ whenever $\epsilon_{1}<\epsilon_{2}$, so that $\lim_{\epsilon ↓0}\mu \left(U_{k}\left(\epsilon \right)\right)=\mu \left(Q_{k}\right)$. We note that here the L is for Lebesgue, inasmuch as these properties are well known to apply for the normalized Lebesgue measure on a compact subset of Euclidean space.*

**Definition** **4.** *Let $P=\left\{Q_{1},\ldots ,Q_{m}\right\}\in P_{\alpha \beta }\left(X\right)$ and $U\left(P,\epsilon \right)=\{U_{1}\left(\epsilon \right),\ldots ,U_{m}\left(\epsilon \right)\}$ be a corresponding open covering in accordance with Definition 3. Then we say that open covering U(P,ϵ) is ϵ-tight with respect to P.*

The following result is a readily verified consequence of the above definitions.

**Lemma** **1.** *Let $P=\left\{Q_{1},\ldots ,Q_{m}\right\}\in P_{\alpha \beta }\left(X\right)$. Then, for any given integer n>1 and ϵ>0 there exists a δ with 0<δ=δ(ϵ,n)<ϵ such that if $U=U\left(P,\delta \right)=\{U_{1}\left(\delta \right),\ldots ,U_{m}\left(\delta \right)\}$ is δ-tight and (consequently for δ sufficiently small) minimal with respect to P, $U^{k}=U^{k}\left(P,\epsilon \right)={\left\{U_{1}\left(\epsilon \right),\ldots ,U_{m}\left(\epsilon \right)\right\}}^{k}$ is ϵ-tight and minimal with respect to $P^{k}={\left\{Q_{1},\ldots ,Q_{m}\right\}}^{k}$ for all 1≤k≤n.*

#### 3.2.2. The Comparison Theorem

We now assemble some of the properties defined above for use as assumptions in the main theorem of this section.

**Definition** **5.** *The measurable dynamical system $D:=\left(f,X,M,\mu \right)$ is T-compatible if there exists a compact metric topology $T_{d}$ on X such that the following properties obtain: (i) f is continuous with respect to $T_{d}$ (ii) μ is Borel with respect to $T_{d}$; (iii) $P_{\alpha \beta }\left(X\right)\neq ⌀$; and (iv) D is of L-type for $T_{d}.$*

The stage has now been set for the following result on comparative equality.

**Theorem** **3.** *Suppose that the DMDS D is T-compatible with respect to $T_{d}$on X and the following additional properties hold:*

*(E1)*  *There exists a sequence of partitions $\left\{P\left(\alpha_{n},\beta_{n}\right)\right\}$ in $P_{\alpha \beta }\left(X\right)$ such that $P\left(\alpha_{n},\beta_{n}\right)≺P\left(\alpha_{n+1},\beta_{n+1}\right)$ for all n∈N and $\left\{\alpha_{n}\right\}$ and $\left\{\beta_{n}\right\}$ are decreasing sequences converging to zero, which means that $P\left(\alpha_{n},\beta_{n}\right)$ is refining in the sense of (ME6).*
*(E2)*  *Moreover, the sequence in (E1) satisfies the following property: For every increasing sequence of positive integers $\left\{j_{k}\right\}$ there exists a dominating sequence of natural numbers $\{n_{k}\},$ with $n_{k}>j_{k}$ for all k∈N, such that $P^{n_{k}}\left(\alpha_{k},\beta_{k}\right)=\left\{Q_{1(k,n_{k})},\ldots ,Q_{m(k,n_{k})}\right\}$ and*
  (10) $$H\left(P^{n_{k}}\left(\alpha_{k},\beta_{k}\right)\right)=\log\left(m(k,n_{k})\right)-\sigma \left(k,n_{k}\right),$$
  *for all k∈N, where $\sigma (k,n_{k})>0$ is bounded for all $(k,n_{k})\in N\times N.$*

*Then, $h_{T_{d}}\left(f\right)=h_{\mu }\left(f\right).$*

**Proof.**  A key element of our proof is the recognition that (E2) expressed in Equation (10) is tantamount to $P^{n_{k}}\left(\alpha_{k},\beta_{k}\right)$ being nearly equiprobable and that a sufficiently tight open covering U of $P(\alpha_{k},\beta_{k})$ yields a tight open cover $U^{n_{k}}$ with respect to $P^{n_{k}}\left(\alpha_{k},\beta_{k}\right)$ such that $\log N\left(U^{n_{k}}\right)=\log\left(m(k,n_{k})\right)$. Consequently,

$$\frac{\log N\left(U^{n_{k}}\right)}{n_{k}}-\frac{H\left(P^{n_{k}}\left(\alpha_{k},\beta_{k}\right)\right)}{n_{k}}:=\Delta \left(U,k,n_{k}\right)=\frac{\sigma (k,n_{k})}{n_{k}}\to 0\;\mathrm{as}\;n_{k}\to \infty .$$

Now, selecting $\left\{P\left(\alpha_{n},\beta_{n}\right)\right\}$ in $P_{\alpha \beta }\left(X\right)$ as in (E1), it follows from (ME 6) that for every ϵ>0 there exists a natural number l=l(ϵ) so large that for any integer k≥l(ϵ) there is a ${\hat{q}}_{k}\in N$ such that

(11) $$\left|\frac{H\left(P^{q_{k}}\left(\alpha_{k},\beta_{k}\right)\right)}{q_{k}}-h_{\mu }\left(f\right)\right|<\frac{\epsilon }{3}$$

whenever $q_{k}$ is an integer greater than ${\hat{q}}_{k}$. Moreover, (E2) implies that for each k≥l(ϵ) there are infinitely instances of Equation (11), which we refer to as ${\tilde{q}}_{k}$, satisfying

(12) $$\left|\frac{H\left(P^{{\tilde{q}}_{k}}\left(\alpha_{k},\beta_{k}\right)\right)}{{\tilde{q}}_{k}}-h_{\mu }\left(f\right)\right|=\left|\frac{\log\left(m(k,{\tilde{q}}_{k})\right)-\sigma \left(k,{\tilde{q}}_{k}\right)}{{\tilde{q}}_{k}}-h_{\mu }\left(f\right)\right|<\frac{\epsilon }{3}.$$

There is, owing to Lemma 1, a decreasing sequence $\left\{\delta_{n}\right\}$ with $\delta_{n}↓0$ as n→∞ associated to a refining sequence of $\delta_{k}$-tight open coverings $\left\{U_{k}\right\}$ of $\left\{P\left(\alpha_{k},\beta_{k}\right)\right\}$ that is refining in the sense of (TE 6). Furthermore, the compactness of *X*, the continuity of f:X→X, (TE 6), (E 2), Equation (10) and Lemma 1 imply that the $\left\{\delta_{n}\right\}$ for the sequence of tight open coverings can be chosen so that $U_{k}^{{\tilde{q}}_{k}}$ is minimal for $P^{{\tilde{q}}_{k}}\left(\alpha_{k},\beta_{k}\right)$ for some ${\tilde{q}}_{k}$ for which Equation (12) obtains,

(13) $$\left|\frac{\log N\left(U_{k}^{{\tilde{q}}_{k}}\right)}{{\tilde{q}}_{k}}-h_{T_{d}}\left(f\right)\right|<\frac{\epsilon }{3}$$

and

(14) $$\frac{\sigma (k,{\tilde{q}}_{k})}{{\tilde{q}}_{k}}<\frac{\epsilon }{3}.$$

Accordingly it follows from Equations (12)–(14) that

$$h_{T_{d}}\left(f\right)-h_{\mu }\left(f\right)=\left|h_{T_{d}}\left(f\right)-h_{\mu }\left(f\right)\right|$$

$$\begin{array}{cc} \; & =\left|\left(h_{T_{d}}\left(f\right)-\frac{\log N\left(U_{k}^{{\tilde{q}}_{k}}\right)}{{\tilde{q}}_{k}}\right)+\left(\frac{\log N\left(U_{k}^{{\tilde{q}}_{k}}\right)}{{\tilde{q}}_{k}}-\frac{H\left(P^{{\tilde{q}}_{k}}\left(\alpha_{k},\beta_{k}\right)\right)}{{\tilde{q}}_{k}}\right)+\left(\frac{H\left(P^{{\tilde{q}}_{k}}\left(\alpha_{k},\beta_{k}\right)\right)}{{\tilde{q}}_{k}}-h_{\mu }\left(f\right)\right)\right|\\ \; & \leq \left|h_{T_{d}}\left(f\right)-\frac{\log N\left(U_{k}^{{\tilde{q}}_{k}}\right)}{{\tilde{q}}_{k}}\right|+\left|\frac{H\left(P^{{\tilde{q}}_{k}}\left(\alpha_{k},\beta_{k}\right)\right)}{{\tilde{q}}_{k}}-h_{\mu }\left(f\right)\right|+\left|\frac{\log N\left(U_{k}^{{\tilde{q}}_{k}}\right)}{{\tilde{q}}_{k}}-\frac{H\left(P^{{\tilde{q}}_{k}}\left(\alpha_{k},\beta_{k}\right)\right)}{{\tilde{q}}_{k}}\right|<\frac{\epsilon }{3}+\frac{\epsilon }{3}+\frac{\epsilon }{3}=\epsilon . \end{array}$$

Hence, as ϵ is arbitrary, the proof is complete. □

We note that it appears that Theorem 2 can also be proved using the concept of an *f*-homogeneous invariant measure introduced in [17]. It is actually likely that the hypotheses in the above theorem are equivalent to Bowen’s *f*-homogeneous invariant Borel probability measure μ property for a discrete dynamical system f:X→X on a compact metric space, which is defined as follows: For every ϵ>0, there exist δ,c>0 such that

$$\mu \left({⋂}_{k=0}^{n-1}f^{-k}\left(B_{\delta }\left(f^{k}\left(y\right)\right)\right)\right)\leq c\mu \left({⋂}_{k=0}^{n-1}f^{-k}\left(B_{\epsilon }\left(f^{k}\left(x\right)\right)\right)\right)$$

for all n≥0 and x,y∈X, where $B_{\delta }$ and $B_{\epsilon }$ are the standard δ and ϵ balls, respectively, for the metric *d* generating the topology $T_{d}$ on *X*. He proved that if this property holds, then

$$h_{T_{d}}\left(f\right)=h_{\mu }\left(f\right)=\lim_{\epsilon \to 0}\left\{\lim\underset{n\to \infty }{\sup}\left[-\frac{1}{n}\log\mu \left({⋂}_{k=0}^{n-1}f^{-k}\left(B_{\epsilon }\left(f^{k}\left(y\right)\right)\right)\right)\right]\right\}$$

In the same vein, it is likely that Theorem 2 can be employed to prove the equivalence of the metric and topological entropies for a DMDS comprising a continuous automorphism of a compact separable topological group leaving the Haar measure invariant. Another thing worth noting is the strong indications that this theorem is apt to have analogs for several variants of entropy, such as relative entropy, as well as some extensions to the noncompact versions of entropy in [18,19,20,21,29,32,33] and these seem like they might be an interesting topics for future research.

### 3.3. Examples of Topological and Metric Entropy Equivalence

We mentioned the case of automorphisms on compact, separable topological groups and now we want to show some examples that follow from Theorems 1 and 2. Most of these examples have already been covered in the literature, so they are mainly meant for illustrative purposes.

**Example** **1.** *Let $f:S^{1}\to S^{1}$ be a rotation that is an irrational multiple of 2π, where $S^{1}$ is the unit circle with the metric d and measure μ both based on the normalized arclength. Then the associated DMDS is ergodic, so Theorem 1 implies that $h_{T_{e}}\left(S^{1}\right)=h_{\mu }\left(S^{1}\right)$, which is readily shown to be zero as is that of every rotation of the circle. It is also a simple matter to obtain the same result using Theorem 2. On the other hand, if we chose a rational multiple of 2π rotation, the DMDS is no longer ergodic, so Theorem 1 cannot be used, but the result can be obtained by employing Theorem 2.*

**Example** **2.** *Consider the standard tent map f=Λ:[0,1]→[0,1] defined as*

$$\Lambda \left(x\right):=\left\{\begin{array}{cc} 2x, & 0\leq x<1/2\\ 2(1-x), & 1/2\leq x\leq 1 \end{array}\right..$$

*combining this with the Lebesgue measure space on the unit interval and the euclidean topology $T_{e}$, we obtain a DMDS on the compact unit interval. It is easy to see that by successively bisecting the unit interval, we obtain a refining sequence of partitions in satisfying Theorem 2 for all $\left\{n_{k}\right\}$, from which we conclude that $h_{T_{e}}\left(\Lambda \right)=h_{\mu }\left(\Lambda \right)=\log2$.*

**Example** **3.** *The same reasoning shows that the 2x(mod1) map $f_{2/1}:\left[0,1\right]\to \left[0,1\right]$ defined as*

$$f_{2/1}\left(x\right):=\left\{\begin{array}{cc} 2x, & 0\leq x<1/2\\ 2x-1, & 1/2\leq x\leq 1 \end{array}\right.,$$

*with respect to the Lebesque measure and the euclidean topology $T_{e}$, is case where $h_{T_{e}}\left(f_{2/1}\right)=h_{\mu }\left(f_{2/1}\right)=\log2$.*

**Example** **4.** *The previous example can be readily generalized to all mx(mod1) maps $f_{m/1}:\left[0,1\right]\to \left[0,1\right]$ for m∈N, m≥2, defined as*

$$f_{m/1}\left(x\right):=\left\{\begin{array}{cc} mx, & 0\leq x<1/m\\ mx-1, & 1/m\leq x<2/m\\ \cdots , & 2/m\leq x<(m-1)/m\\ mx-(m-1) & (m-1)/m\leq x\leq 1 \end{array}\right.,$$

*with the same measure and topological structure above for the 2x(mod1) map. The result is $h_{T_{e}}\left(f_{m/1}\right)=h_{\mu }\left(f_{m/1}\right)=\log m$.*

**Example** **5.** *We can neatly recast the mx(mod1) map examples as smooth maps on a smooth manifold and extend them to higher dimensions with ease. To begin, we again let $S^{1}$ be the unit circle in the complex plane C with the standard arclength measure μ normalized by a factor of 1/2π, so that it is a probability measure, with the usual Riemannian arclength metric d also scaled by a factor of 1/2π. Then, the mx(mod1) maps can be identified with the smooth maps $z\to z^{m}$ restricted to the unit circle; namely, $F_{m}:S^{1}\to S^{1}$ defined by ${\left(z^{m}\right)}_{|S^{1}}$ so that $F_{m}\left(e^{i\theta }\right):=e^{im\theta }$, and this combination defines a DMDS on a compact space. By mimicking the construction in the previous example, it can be readily shown the Theorem 2 implies that $h_{T_{e}}\left(F_{m}\right)=h_{\mu }\left(F_{m}\right)=\log m$. Similarly, given an n-tuple $m=\left(m_{1},\ldots ,m_{n}\right)\in N^{n},$ we define the smooth self map of the n-torus $\Phi_{m}:T^{n}\to T^{n}$ by*

$$\Phi_{m}\left(e^{i\theta_{1}},\ldots ,e^{i\theta_{m}}\right):=\left(e^{im_{1}\theta_{1}},\ldots ,e^{im_{n}\theta_{n}}\right),$$

*where as usual, $T^{n}$ is just the cartesian product of n copies of the unit circle with the product topology and product measure associated to the single circle. This then comprises a smooth DMDS on the compact n-torus, which can be easily shown to satisfy the hypotheses of Theorem 2 and yield $h_{T_{e}}\left(\Phi_{m}\right)=h_{\mu }\left(\Phi_{m}\right)=\log\left(m_{1}\cdots m_{n}\right)$.*

## 4. Shannon Entropy as a Special Case of Metric Entropy

We shall prove that Shannon’s entropy may be formulated in the context of a Bernoulli scheme; whence, it is equal to the corresponding metric entropy owing to the Kolmogorov–Sinai entropy theorem [23,25], which is also covered in [12,13,15,24]. What we undertake in this section was more or less observed in the process of the development of K–S entropy, although not actually proved in detail as in what follows. In this regard, the work of Frigg [34] should also be noted. To recast Shannon entropy (described above) as a measurable dynamical system called a *Bernoulli scheme* or *Bernoulli shift*, we define the phase space *X* as the set of all bi-infinite sequences of symbols; namely,

$$X:=S^{Z}:=\left\{\zeta :\zeta :Z\to S\right\}=\left\{\left(\ldots ,\zeta_{-2},\zeta_{-1},\zeta_{0}.\zeta_{1},\zeta_{2},\ldots \right):\zeta_{k}\in S=\left\{s_{1},\ldots ,s_{n}\right\}\;\;\forall k\in Z\right\},$$

and the (bijective) map s:X→X to be the (Bernoulli) shift

$$s\left(\zeta \right)\left(k\right)=\zeta \left(k+1\right)$$

for all k∈Z together with the following probability distribution $\left(p_{1},\ldots ,p_{n}\right)$ for the symbol set *S*, where $p_{k}:=p\left(s_{k}\right)>0$ for all k∈Z and $p_{1}+p_{2}+\cdots +p_{n}=1$. This definition fits rather nicely with the information foundation of Shannon entropy inasmuch as it corresponds to reading the string of symbols from left to right one at a time.

To complete the reformulation as a measurable dynamical system, it remains to define a σ-algebra of μ-measurable subsets of *X*, denoted as M, where μ is an s-invariant probability measure on *X*. This can be done in many ways, so the trick is to find a definition that yields a metric entropy equal to the Shannon entropy. Toward this end, we start by defining *cylinder sets* of the form

$$C\left(F,\psi \right):=\left\{\zeta \in X:\zeta_{|F}=\psi \right\},$$

where *F* is a finite set of integers, $\psi \in S^{F}$ and $\zeta_{|F}$ is the restriction of ζ to F⊂Z, and let C be the collection of all cylinder sets together with the null set and all of *X*.

Next, we introduce the set function $\mu_{0}:C\to R$ defined as follows:

$$\mu_{0}\left(E\right):=\left\{\begin{array}{cc} 0, & E=⌀\\ 1, & E=X\\ \prod_{k\in F}p\left(\psi (k)\right), & E=C\left(F,\psi \right) \end{array}\right..$$

Now, there is a theorem of Kolmogorov [22] (see also, for example [24], p. 628) of a rather technical nature stating that the above set function can be uniquely extended to a complete probability measure μ:M→R, called the *product measure determined by the distribution*$\left(p_{1},\ldots ,p_{n}\right)$, where M is the smallest σ-algebra containing C and $p_{k}:=p\left(s_{k}\right)$ for all 1≤k≤n.

Finally, to show that this measure is invariant under the shift map, it suffices to verify this for cylinder sets, and this can readily seen by first observing that

$$s^{-1}\left(C\left(F,\psi \right)\right)=C\left(\tilde{F},\tilde{\psi }\right),$$

where $\tilde{F}:=F+1:=\left\{k+1:k\in F\right\}$ and $\tilde{\psi }:\tilde{F}\to S$ with $\tilde{\psi }\left(k+1\right)=\psi \left(k\right)$ for all k∈F. Consequently,

$$\mu \left(s^{-1}\left(C\left(F,\psi \right)\right)\right)=\mu \left(C\left(\tilde{F},\tilde{\psi }\right)\right)=\prod_{j\in \tilde{F}}\mu_{0}\left(\tilde{\psi }\left(j\right)\right)=\prod_{k\in F}\mu_{0}\left(\psi (k)\right)=\mu \left(C\left(F,\psi \right)\right).$$

Thus we have reformulated the Shannon communication system with alphabet *S* as the measurable dynamical system $\left(s,X:=S^{Z},M,\mu \right)$, where M and μ are as defined above, which is called a Bernoulli scheme and often denoted as $B\left(p_{1},\ldots ,p_{n}\right)$. The details necessary to prove that the metric entropy of the Bernoulli scheme equals the Shannon entropy of the corresponding communication system are quite extensive and covered rather neatly and completely in [24], so we shall only try to summarize the results in what we hope is a readily understood fashion, at least from an intuitive standpoint.

First, the Kolmogorov–Sinai entropy theorem can be roughly stated as follows: *Let*$D=\left(f,X,M,\mu \right)$*be a measurable dynamical system withfinvertible, as it is in the case of*$B\left(p_{1},\ldots ,p_{n}\right)$*, such that there is a measurable partition*P*for which*M*is the smallest*σ*-algebra containing the union of the unions of all sets of the form*

$$f^{-m}P\vee f^{-m+1}P\vee \cdots \vee f^{m-1}P\vee f^{m}P,$$

*as* *m* *ranges over the natural numbers* N. *Then* $h_{\mu }\left(f\right)=H\left(P,f\right).$

Now, it is not difficult to show (see, e.g., [24]) that the measurable partition

$$P:=\left\{C_{k}:1\leq k\leq n\right\},$$

where $C_{k}:=$$\left\{\zeta \in X=S^{Z}:\zeta_{0}=s_{k}\right\}$, satisfies the requirements of the Kolmogorov–Sinai theorem. This follows primarily from the fact that $s^{-m}\left(C_{k}\right)=\left\{\zeta \in X:\zeta_{m}=s_{k}\right\}$ for all m∈N. Now the entropy of the partition P is

$$H\left(P\right)=-\sum_{k=1}^{n}\mu \left(C_{k}\right)\log\mu \left(C_{k}\right)=-\sum_{k=1}^{n}p_{k}\log p_{k}.$$

Moreover, we find that

$$H\left(P^{m}\right)=-\sum_{k_{0},\ldots ,k_{m-1}}^{n}\prod_{j=0}^{m-1}p_{k_{j}}\log\left(\prod_{j=0}^{m-1}p_{k_{j}}\right)=-m\sum_{k=1}^{n}p_{k}\log p_{k}.$$

Therefore, it follows from the two expressions above and the entropy theorem of Kolmogorov–Sinai that

$$h_{\mu }\left(s\right)=H\left(P,s\right)=\lim_{m\to \infty }\frac{H\left(P^{m}\right)}{m}=-\sum_{k=1}^{n}p_{k}\log p_{k},$$

which is the desired result.

### A Simple Bernoulli Scheme Example

To illustrate the result just obtained, we shall verify it by direct calculation for a binary Bernoulli scheme. In particular, consider $X:=B(p_{1},p_{2})$, with S:={0,1}, in which case it follows readily from the definitions above that the phase space of the dynamical system comprises all bi-infinite binary sequences; namely,

$$X=2^{Z}=\left\{\zeta :\zeta :Z\to \{0,1\}\right\}=\left\{\cdots a_{-2}a_{-1}a_{0}.a_{1}a_{2}a_{3}\cdots :a_{k}\in \left\{0,1\right\}\;\forall k\in Z\right\},$$

and the map s:X→X is the shift map defined as s(ζ)(k):=ζ(k+1) or equivalently

$$s\left(\cdots a_{-2}a_{-1}a_{0}.a_{1}a_{2}a_{3}\cdots \right):=\cdots a_{-2}a_{-1}a_{0}a_{1}.a_{2}a_{3}\cdots .$$

We know from the above that for the measurable partition

$$P:=\left\{C_{0}:=C\left(\{0\},0\right),C_{1}:=C\left(\{0\},1\right)\right\},$$

where $C_{k}:=$$\left\{\zeta \in X={\left\{0,1\right\}}^{Z}:\zeta_{0}=k\right\}$ for k=0,1, has the following entropy

$$H\left(P\right)=-p_{1}\log p_{1}-p_{2}\log p_{2}.$$

Our intention is to show that this is equal to the entropy with respect to the shift map s, that is

$$h_{\mu }\left(s\right)=H\left(P\right).$$

Owing to the readily verified fact that every member of ${⋁}_{k=-m}^{m}s^{-k}P$ is of the form $C\left(Z_{-m}^{m},\varphi \right)$ with $\varphi :Z_{-m}^{m}\to \left\{0,1\right\}$ and the Kolmogorov–Sinai entropy theorem (see e.g., [24]), it suffices to prove that every cylinder $C\left(F,\psi \right)$ satisfies

$$C\left(F,\psi \right)\subset ⋃\left\{C\left(Z_{-m}^{m},\varphi \right):\varphi :Z_{-m}^{m}\to \left\{0,1\right\}\right\},$$

where $Z_{-m}^{m}:=\left\{l\in Z:\left|l\right|\leq m,m\in N\right\}$. This is obvious since *F* is finite, which proves the desired result.

## 5. Concluding Remarks

After a brief review of the main features and fundamental properties of topological, metric (Kolmogorov–Sinai) and Shannon entropy, we embarked on our effort to establish dynamical systems theory as their principal connective thread. We began with comparisons between the topological entropy, which may be considered the most general manifestation of the multifarious forms, and the metric entropy, perhaps the most real- world applicable of the embodiments of entropy. For example, we recounted the variational principle that the topological entropy of a discrete dynamical system is the supremum of the metric entropies over all possible corresponding discrete measurable dynamical dynamical systems, which naturally leads to the question of whether or not the supremum is actually assumed. In that vein, we proved our main theorem giving sufficient conditions for the equality of the topological and Komolgorov–Sinai entropies and provided several illustrative examples. Finally, we showed that Shannon’s information entropy is a special case of metric entropy, inasmuch the a dynamical information system can be identified with a Bernoulli scheme for which the Kolmogorov–Sinai entropy theorem provides a formula for the entropy identical to that of Shannon. In summary then, our project aimed at providing a rigorous underlying dynamical system theme for several of the more important entropy definitions.
